# Evidence of Decoupling Protein Structure from Spidroin Expression in Spider Dragline Silks

**DOI:** 10.3390/ijms17081294

**Published:** 2016-08-09

**Authors:** Sean J. Blamires, Michael M. Kasumovic, I-Min Tso, Penny J. Martens, James M. Hook, Aditya Rawal

**Affiliations:** 1 Evolution & Ecology Research Centre, School of Biological, Earth & Environmental Sciences, University of New South Wales, Sydney 2052, Australia; m.kasumovic@unsw.edu.au; 2 Department of Life Science, Tunghai University, Taichung 40704, Taiwan; spider@thu.edu.tw; 3 Graduate School of Biomedical Engineering, University of New South Wales, Sydney 2052, Australia; p.martens@unsw.edu.au; 4 NMR Facility, Mark Wainwright Analytical Centre, University of New South Wales, Sydney 2052, Australia; j.hook@unsw.edu.au (J.M.H.); a.rawal@unsw.edu.au (A.R.)

**Keywords:** amino acids, high performance liquid chromatography, mechanical properties, orb weaving spiders, protein secondary structures, silk spinning, solid-state nuclear magnetic resonance spectroscopy

## Abstract

The exceptional strength and extensibility of spider dragline silk have been thought to be facilitated by two spidroins, major ampullate spidroin 1 (MaSp1) and major ampullate spidroin 2 (MaSp2), under the assumption that protein secondary structures are coupled with the expressed spidroins. We tested this assumption for the dragline silk of three co-existing Australian spiders, *Argiope keyserlingi*, *Latrodectus hasselti* and *Nephila plumipes*. We found that silk amino acid compositions did not differ among spiders collected in May. We extended these analyses temporally and found the amino acid compositions of *A. keyserlingi* silks to differ when collected in May compared to November, while those of *L. hasselti* did not. To ascertain whether their secondary structures were decoupled from spidroin expression, we performed solid-state nuclear magnetic resonance spectroscopy (NMR) analysis on the silks of all spiders collected in May. We found the distribution of alanine toward β-sheet and _3,10_helix/random coil conformations differed between species, as did their relative crystallinities, with *A. keyserlingi* having the greatest _3,10_helix/random coil composition and *N. plumipes* the greatest crystallinity. The protein secondary structures correlated with the mechanical properties for each of the silks better than the amino acid compositions. Our findings suggested that a differential distribution of alanine during spinning could decouple secondary structures from spidroin expression ensuring that silks of desirable mechanical properties are consistently produced. Alternative explanations include the possibility that other spidroins were incorporated into some silks.

## 1. Introduction

The exceptional strength, extensibility and toughness of spider dragline, or major ampullate (MA), silk make it a desirable material for multiple industrial uses [1]. Dragline silk is traditionally thought to be comprised of two proteins (spidroins), conventionally called major ampullate spidroin 1 (MaSp1) and major ampullate spidroin 2 (MaSp2). Generally, the structures of these proteins are considered critical to the mechanical function of dragline silk.

Techniques that can probe protein structures include small and wide angle X-ray scattering, Fourier Transform Infrared Spectroscopy (FTIR) and various forms of nuclear magnetic resonance (NMR) spectroscopy [2,3,4,5,6,7]. Small and wide angle X-ray scattering are used explicitly for examining the size, density and orientation of crystalline and non-crystalline structures [5]. Since NMR detects magnetically active isotopes such as ^1^H, ^13^C and ^15^N, it is used to identify the molecular orientations and bonding arrangements within protein secondary structures [8]. As such, solid state nuclear magnetic resonance (ssNMR) spectroscopy has been effectively used to assess the relationship between spider dragline silk amino acid compositions and the formation of protein secondary structures, as well as protein chain dynamics [7,8,9,10,11,12,13,14,15].

ssNMR has shown MaSp1 to consist of multiple (GA)_n_, (GGX)_n_ and (A)_n_ repeated amino acid sequences (G = glycine, A = alanine and X = other amino acids) and it is assumed that these sequences promote the formation of crystalline β-sheet structures in assembled fibers [3,9,16]. MaSp2, on the other hand, consists of multiple (GPGXX)_n_ repeated sequences (P = proline) [10,17]. This sequence is currently assumed to self-assemble into β-spirals and type-II β-turns in the silk fibers [6,18]. Since MaSp2 contains (GPGXX)_n_ repeated sequences, the proline composition of dragline silk has been used as an indicator of the presence of MaSp2. Thus, the combined expression of MaSp1 and MaSp2 are assumed to be coupled to the presence of various quantities of crystalline β-sheets, β-spirals and type-II β-turns, which are in turn thought to provide dragline silk with its great strength and extensibility [1].

The positioning of the alanine residues is an important indicator of dragline silk structure that is detectable using ssNMR [13,19,20,21]. Generally, the majority (>80%) of the alanine in MaSp1 resides in β-sheets, predominantly as (A)_n_ repeated sequences [13,19,22,23]. The rest of the MaSp1 alanine lies in α-helices, _3,10_helices and type-II β-turns [23]. The distribution of alanine in MaSp2 varies substantially among silks from different spider species [13,21,22,23]. Since the C=O segments bond weakly with adjacent amine segments [24], alanine is mobile during spinning and this mobility is identifiable using ssNMR techniques [19]. Hence the repositioning of alanine within the spidroins might be a detectable mechanism influencing protein secondary structures within dragline silk fibers.

Glycine residues are, likewise, important identifiers of dragline silk structure when using ssNMR. In MaSp1 glycine primarily forms either (GA)_n_, repeating sequences within β-sheets or (GGX)_n_ repeating sequences within GlyII-helices [25]. Glycine may be mobile during spinning, albeit to a lesser extent than alanine, so variability in glycine distribution between individual dragline silks might also be expected. Proline is found within the (GPGXX)_n_ repeating sequences of MaSp2 and is identified using ssNMR by its tendency to form kinks [18]. It is thought to facilitate extensibility by forming weak hydrogen bonding between the structural components that enable the chains to freely slide past each other [26,27,28,29].

ssNMR studies of dragline silk have to date mostly focused on elucidating the amino acid compositions, secondary structures, and molecular dynamics of the spidroins from a few model species, such as *Nephila clavipes*, *Latrodectus hesperus* and *Argiope aurantia* [17,23,25,30]. One study [13] used ssNMR to compare the structures and properties of dragline silks from a range of species from four different spider genera: *Araneus*, *Argiope*, *Nephila* and *Latrodectus*. Among these genera *Araneus* and *Argiope* had dragline silks high in MaSp2 compositions and proline molar compositions of ~11%–14%, while *Nephila* and *Latrodectus* were low in MaSp2 and proline compositions of only ~1%–2% [13].

Studies comparing amino acid compositions in the dragline silks of different species [16,27,31,32,33] have revealed similar dichotomies in silks produced among different spiders, i.e., spiders seem to produce silks that are either high in MaSp2 or relatively devoid of MaSp2. There, however, does not seem to be any phylogenetic relationship between the spiders expressing silks that are relatively high or low in MaSp2 [32,33]. An explanatory hypothesis for the apparent dichotomy in dragline silk spidroin expression among spiders is that factors such as ecological circumstances, or spider body size, condition, or aging affect spidroin expression. For instance, MaSp2 expression may be down-regulated when a spider is starved or deprived of nutrients [31,34,35]. The finding that silk amino acid composition can differ in the dragline silks from the same species of spider from different regions [36,37] also supports this prediction. Nevertheless, the mechanical performance of a spider’s silk may be unaffected by variations in spidroin expression [33,38,39,40]. Thus, there seem to be several ways by which variations in silk mechanical property can be induced among different spiders without sacrificing functional effectiveness.

As silk flows through the major ampullate gland as a crystalline liquid the actions of pH change, salt concentrations and shear forces in the duct effect the formation of the secondary structures [41,42,43,44,45]. Accordingly, a possible reason why the mechanical properties of dragline silk sometimes vary independent of spidroin expression is that the silk spinning processes decouple spidroin expression from secondary structure formation through an “on-the-fly” redistribution of alanine or glycine residues [46]. This explanation nonetheless remains to be empirically tested in different spiders using multiple techniques. We therefore determined herein the spidroin expression, protein secondary structure and mechanical properties of the dragline silks of three co-existing species of spider from Sydney, Australia: *Argiope keyserlingi*, *Nephila plumipes* and *Latrodectus hasselti* using high performance liquid chromatography (HPLC), ssNMR and tensile testing techniques. All of their silks were collected in May 2014. We determined the structural formations induced by the alanine and glycine residues in the silks from the ssNMR measurements. Any incongruencies between amino acid compositions, protein secondary structures and mechanical properties among spiders were construed as indicating that spinning processes decoupled spidroin expression from protein secondary structures and, thus, mechanical properties [44,45]. We additionally investigated temporal variations in spidroin expression by performing further HPLC analyses on *Argiope keyserlingi* and *Latrodectus hasselti* silks collected in November. We regarded any similarities in amino acid composition between species at any particular time of year or any differences within species at different times of year as indicating that ecological circumstances or demographic factors influenced spidroin expressions.

## 2. Results and Discussion

### 2.1. Amino Acid Compositions

We found that the dragline silk amino acid compositions did not differ between the three species examined in May (MANOVA: Wilk’s *λ* = 0.161; *F* = 2.387; *df* = 10, 16; *p* = 0.058) (Table 1a). We then expanded our spidroin expression analysis by comparing the amino acid compositions of *Latrodectus hasselti* and *Argiope keyserlingi* silks collected in November and found differences between species (MANOVA: Wilk’s *λ* = 0.022; *F* = 25.670; *df* = 12,52; *p* < 0.001) (Table 1b). Furthermore, the amino acid composition of *A. keyserlingi* dragline silk collected in May differed from that collected in November (MANOVA: Wilk’s *λ* = 0.048; *F* = 15.99; *df* = 5,4; *p* = 0.009), with the silks from spiders collected in November having a greater percentage alanine (*p* = 0.038) and proline (*p* < 0.001) than those collected in May. On the other hand the amino acid composition of *L. hasselti* dragline silk did not differ between May and November (MANOVA: Wilk’s *λ* = 0.313; *F* = 1.578; *df* = 5, 4; *p* = 0.302). By comparing the compositions attained here with those derived from *Latrodectus hesperus* [47] and *Argiope bruennichi* [48] dragline silk gene sequences (Table 1c), our results suggested that each of the spiders produced dragline silk comprising of primarily MaSp1 in May, while in November *A. keyserlingi* produced dragline silk that likely comprised of a greater proportion of MaSp2. Thus, we concluded that spidroin expression in the dragline silk of *A. keyserlingi* differs at different times of year, possibly as a result of changing ecological or demographic circumstances.

We found less variability around the mean compositions for silk collected in May than for silks collected in November (see Table 1). Work and Young [49] noted that some segments within individual silk fibers could differ in amino acid composition and suggested that within a single fiber there may be differences in the ratio of MaSp1 and MaSp2. We accounted for such within fiber variability in amino acid composition here by analyzing the amino acid composition of entire dragline threads from each individual of each species. Accordingly, we considered this source of variation as not being responsible for any of the variation in dragline silk amino acid compositions between species or within species at different times of year. The spiders that we used were all found in similar urban habitats in Sydney, Australia. *L. hasselti*, however, was found in more sheltered microhabitats than *A. keyserlingi* (*L. hasselti* was mostly found underneath rigid structures, e.g., park benches, whereas *A. keyserlingi* was more frequently found exposed among vegetation). Accordingly, it seems that the amino acid composition of *A. keyserlingi* silk could vary in concordance with relatively small-scale environmental fluctuations.

Another possibility for the fluctuations in amino acid composition in *A. keyserlingi* silk is that MaSp1 and MaSp2 are not the only spidroins being expressed. Recent proteomic data using mass spectrometry of solubilized dragline silk from *L. hesperus* followed by in-solution tryptic cleavage supported the presence of another spidroin, AcSp1 (acinform spidroin 1), within the major ampullate gland as well as in dragline silk fibers [50]. The presence of AcSp1 might explain the unusually high serine compositions in the dragline silks of all species, the lower than predicted (based on sequences of *A. bruennichi* dragline silk [48]) glycine compositions in *A. keyserlingi*’s dragline silk in May and November, and the low alanine composition of *L. hasselti’*s dragline silk (compared to that of *L. hesperus* [47]) in May (see Table 1).

We found a seasonal difference in spidroin expression in *A. keyserlingi* dragline silk but an absence of any change in the composition in *L. hasselti* dragline silk, which is consistent with studies showing that amino acid compositions in spider dragline silks that are relatively low in MaSp2 vary less extensively across environments than those high in MaSp2 [31,32,35]. We controlled for diet and nutritional effects on spidroin expression here by pre-feeding the spiders a standardized solution so recent diet did not affect dragline spidroin expression in any species at any time. A likely explanation for the seasonal variation in the amino acid composition of *A. keyserlingi* dragline silk is that MaSp2 was considerably down-regulated in May because it is synthesized at a considerable metabolic expense [34,35]. MaSp1 synthesis, on the other hand, is significantly less expensive, so is largely unregulated [31]. The three species examined here have different seasonal activity patterns, so their MaSp2 expression is likely to be regulated differently. Studies incorporating more environmental measurements across seasons and habitats are nonetheless required to better elucidate the particular circumstances that induce changes in dragline silk spidroin expression among co-existing web building spiders.

### 2.2. Solid-State Nuclear Magnetic Resonance Spectroscopy

For ^13^C cross -polarization magic-angle spin (CPMAS) NMR experiments the ^13^C signal of a given functional group is dependent on the ^1^H–^13^C cross-polarization dynamics. The ^13^C signal intensity in a CPMAS spectrum will depend upon: (a) the protonation state of the carbon; (b) the local molecular mobility of the ^13^C site; and (c) the concentration of the ^13^C species in the material. Since the signals for the alanine and glycine functional groups were constant among the three spider’s silk samples here and we compared the same kind of ^13^C species, i.e., the methyl species, our spectra depended only on local molecular motions and concentration. We had similar materials in all samples, i.e., large spidroins, so any difference in the local molecular motions will be a function of the degree of hydration. Although we were careful not to expose the silks to the atmosphere, we took the additional precaution of measuring the ^1^H spectra of the different silks. While the signals from the water peak (5–10 ppm [21]) indicated that some water binding was present, the signals were generally broad and the peaks relatively small (see Figure S1) compared to what would be expected if the material had absorbed a large amount of water, for instance during supercontraction [11,19]. Thus, we were sure that humidity did not strongly influence our spectra attained. The tall, narrow peaks associated with each of the curves of Figure S1 were all found around 0 ppm so attributed to large amino acids or molecules associated with the silk’s skin [6,8]. Subsequently, we were confident that we attained highly reliable ^13^C CPMAS NMR spectra, enabling us to compare the relative intensities of the ^13^C signals among the different dragline silks and to compare secondary structures between individuals and species.

**Figure 1:**
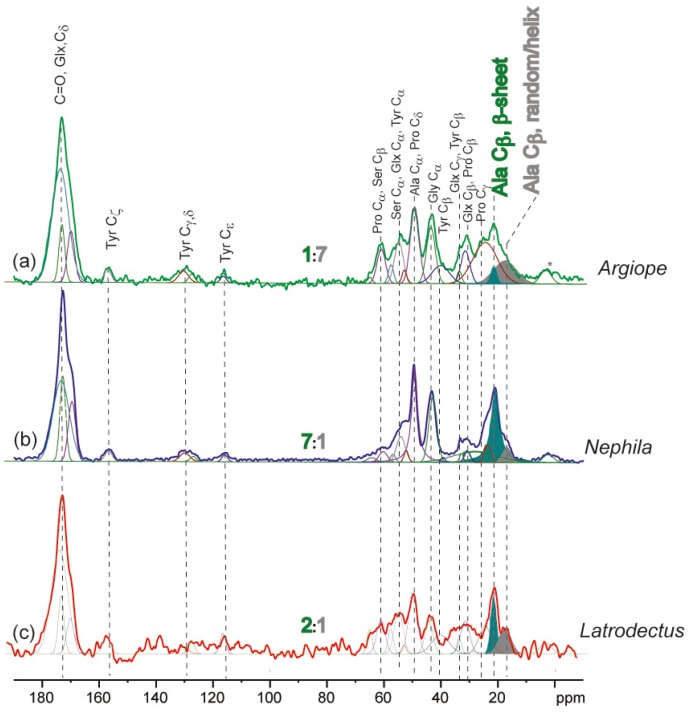
^13^C CPMAS ssNMR of silk from individual: (**a**) *Argiope keyserlingi* (*Argiope*); (**b**) *Nephila plumipes* (*Nephila*); and (**c**) *Latrodectus hasselti* (*Latrodectus*). The spectral peaks shaded green represent poly-alanine β-sheets, while those shaded grey represent poly-alanine _3,10_helices/random coils. Accordingly, the spectra show that there are differences in the ratios of alanine in β-sheets: alanine in _3,10_helices/random coil between *A. keyserlingi* (1:7), *N. plumipes* (7:1) and *L. hasselti* (2:1) silks*.* Spectra of individual spiders within each species are shown in the Supplementary Materials (Figure S1).

The ^13^C cross-polarization magic-angle spectra for the three species’ silks are presented in Figure 1. All spectra were scaled to equal the intensity of the C=O peak. An additional plot of the variation of the spider silk among individuals of each species is presented in the Supplementary Materials (Figure S2). The peaks were assigned in accordance with Creager et al. [13] to enable estimation of the different residues and domains. The most striking difference that our ssNMR analyses found between the spidroin structures of the three species silks collected in May was the relative intensities of the C_β_ signal on alanine in β-sheets (peaking at 21.5 ppm) versus _3,10_helices/random coil (peaking at 17.5 ppm). We found that *A. keyserlingi* silk collected in May had the lowest β-sheet signal intensities relative to that of _3,10_helices/random coil, while the *N. plumipes* and *L. hasselti* silk collected in May had much stronger β-sheet signal intensities relative to _3,10_helices/random coil (see Figure 1). Assuming similar cross polarization of the methyl sites in the alanine β-sheet or _3,10_helices/random coil conformations, we estimated that the ratio of alanine in β-sheets: Alanine in _3,10_helices/random coil is 1:7 in *A. keyserlingi* silk and 7:1 in *N. plumipes* silk and 2:1 in *L. hasselti* silk*.* Thus, while the amino acid compositions suggested that all of the spiders produced dragline silk comprising of primarily MaSp1 in May, ssNMR revealed that the secondary structures of the proteins within each species’ dragline silk differed profoundly.

While *L. hasselti* dragline silk was structurally more similar to *N. plumipes’* silk than *A. keyserlingi’*s silk, there was more structural variation between individual fibers in this species compared to the other two species (Figure S2). This may be because there were considerably high signal-to-noise ratios in the resonances attained for the *L. hasselti* silks because of their exceptional thinness (<2 µm). On the other hand spinning conditions within the glands of individual *L. hasselti* may have induced a wider array of structural variations among the individual dragline silks, or the presence of AcSp1 in *L. hasselti* dragline silks was variable among individuals.

The secondary structures within *A. keyserlingi* dragline silk resembled those expected for MaSp2 predominant silk, while those within *N.* *plumipes* and *L. hasselti* dragline silk more resembled those expected for MaSp1 predominant silk. *N. plumipes*’ dragline silk yielded the narrowest signal at the alanine C_α_ site (70 ppm), indicating a higher degree of order and crystallinity in their silks compared to *A. keyserlingi* or *L. hasselti* silks. Since there were incongruencies between the amino acid compositions revealed by HPLC and the protein secondary structures revealed by ssNMR we concluded that silk spinning processes may have acted to dissociate the protein secondary structures from spidroin expression, or other spidroins may have been incorporated into some of the silks.

### 2.3. Influences Affecting Mechanical Properties

Representative stress–strain curves for three individual *L. hasselti*, *A. keyseringi* and *N. plumipes* silks are shown in the Supplementary Materials (Figure S3). One feature of the curves is what appears to be slippage of the fibers at the early stages of extension. We expect that this was a by-product of the low resolution of measurements of our testing machine, which we found to show high variability when measuring some exceptionally fine fibers. Our procedures, nevertheless, were consistent for all of the silks analyzed and the results were detailed enough to make comparisons between species.

We found that the native state silks of each species had significantly greater ultimate strength but were less extensible than their supercontracted silks, indicating that alignment of the crystalline and amorphous region proteins affected the mechanical properties of each species’ native silks independent of spidroin expression [38,51]. We suggest that studies involving intraspecific comparisons require more precise mechanical testing methods.

**Figure 2:**
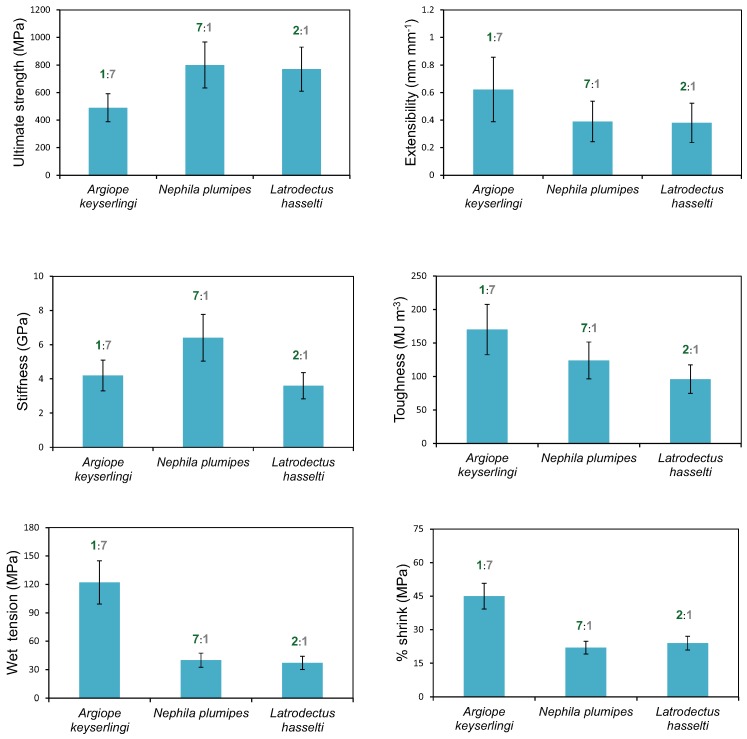
Comparisons of the ultimate strength, extensibility, stiffness and toughness of native silks, and wet tension and percent shrink of supercontracted silks of *L. hasselti*, *A. keyserlingi* and *N. plumpes* dragline silks taken from spiders collected in May. Numbers associated with the respective species’ bars show the β-sheets (green): _3,10_helices/random coil (grey) ratios ascertained by ssNMR (see Figure 1).

The mechanical properties of the dragline silks from all three species differed (Figure 2). These differences reconciled well with our ssNMR structural analysis. For instance, we found *N. plumipes* silk to have the highest ultimate strength, which can be attributed to the high crystallinity of its silk. On the other hand, *A. keyserlingi*’s silk had the greatest toughness and extensibility, properties that can be attributed to its low crystallinity and greater distribution of alanine toward _3,10_helices/random coils rather than β-sheets. Moreover, despite having similar amino acid compositions to the other two species *A. keyserlingi* silk had the greatest percent shrinkage, which may be attributable to its high proportion of helical/coiled structures, as these structures are considered more amenable to extension under tension compared to the crystalline structures [45,52,53]. The theoretical premise for this prediction is that the weak hydrogen bonds between the amino acid residues in random coil structures would likely have been accessed more readily by water resulting in loss of alignment in the amorphous region [12,28,29,54].

The amino acid composition of *A. keyserlingi* dragline silk collected in May did not differ from the silk amino acid compositions of the other two species, so spidroin expression does not explain the variations in mechanical properties found between the three species’ silks. We therefore deduced that the incongruencies between the amino acid compositions and protein secondary structures and mechanical properties among the three species is evidence of decoupling between silk protein secondary structures and spidroin expression. Glandular processes that might induce decoupling may include the shear forces experienced during the final phase of silk spinning inducing crystalline and amorphous region self-alignment in the spun silk [44,45,46]. Additional amorphous region alignment may be extenuated at the valve by the influence of friction during drawing [45,55]. These actions could explain why the mechanical properties of the silks in all three species varied independent of their spidroin expressions and why *A. keyserlingi*’s dragline silk in May resembled MaSp1 predominant silk in amino acid composition but resembled a MaSp2 predominant silk in secondary structure. An alternative possibility is that additional spidroins, such as AcSp1 [50], were additionally expressed in *A. keyserlingi* dragline silk, rendering the influence of amino acid composition on mechanical properties inconsistent with that expected if only MaSp1 and MaSp2 were expressed.

## 3. Experimental Section

### 3.1. Spiders and Silk Collecting

We collected five adult females of three species, *Argiope keyserlingi*, *Nephila plumipes* and *Latrodectus hasselti*, from similar urban habitats in Sydney, New South Wales, Australia, in May 2014, as at this time all three species were active. We collected a further 5 *L. hesselti* and 5 *A. keyserlingi* in November 2014 (the time of year when the activities of these two spiders peaked) to determine whether spidroin expression varied in similar spiders at different times of year. *N. plumipes* was not collected in November as it was not active at this time.

Upon collection of the spiders, we measured their body length to ±0.1 mm using digital Vernier calipers (Caliper Technologies Corp., Mountain View, CA, USA) and their mass to ±0.001 g using an electronic balance (Ohaus Corp., Pine Brook, NY, USA) before placing them in 115 mm (wide) × 45 mm (high) plastic circular containers. The containers had perforated wire mesh lids with a 20 mm long slit cut into them using a Stanley knife to facilitate feeding with a 50 μL micropipette. We fed all of the spiders 20 μL (*L. hesselti* and *A. keyserlingi*) or 50 μL (*N. plumipes*) of an unlabeled 30% *w*/*v* glucose solution daily over five days (for details of solution preparation see Blamires et al. [31,38]) to standardize the recent diet of all spiders prior to collecting their silk. We reweighed the spiders after the 5 days of feeding and individuals whose mass deviated >50% from the mean for the species (one *L. hasselti* and one *N. plumpes* collected in May) were discarded.

We anaesthetized each spider using CO_2_ gas and carefully pulled a single dragline fiber from the spinnerets using tweezers and wrapped it around a glass tube connected to a mechanical spool. The spool was spun at a constant speed (1 m·min^−1^) for ~1 h whereupon 10–15 mg of silk was collected. We estimated that this amount of silk closely represents the complete store of silk from the major ampullate gland for each of these species. All silks were extracted under controlled temperature (~25 °C) and humidity (~50% R.H.) in still air, so reeling and the post-spin environment did not influence their subsequent chemical or mechanical properties.

### 3.2. High Performance Liquid Chromatography (HPLC)

We used HPLC to determine amino acid compositions using 1–5 mg of silk collected from the 13 individual spiders (4 or 5 individual × 3 species) collected in May and 1–5 mg of silk from the 10 (five *L. hasselti* and five *A. keyserlingi*) spiders collected in November. Thus, 25 samples were prepared for HPLC analysis in total.

We weighed all of the silk samples to the nearest 0.001 mg on an electronic balance, before placing them into 100-µL Eppendorf tubes, submerging them in 99% hexaflouro-isopropanol solvent (500 µL of per mg of silk) and leaving them overnight. After removal of the solvent, the samples were then placed in glass tubes and hydrolyzed in 6 M HCl for 24 h in a furnace at 115 °C. The amino acids were separated in a Pico-Tag amino acids column (Waters, Milford CA, USA) and we determined the mole percentages of glutamine, serine, proline, glycine, and alanine (as these amino acids represent >80% of the total amino acids of dragline silks in most spiders [56]).

We used a single-factor multivariate analysis of variance (MANOVA) and Fisher’s Least Significant Difference post-hoc analysis to compare the mean (±standard errors) mole percentages of glutamine, serine, proline, glycine, and alanine between species when collected in May. We additionally performed between species (*L. hasselti* versus *A. keyserlingi*) comparisons of the amino acid mole percentages for silks collected in May and November, as well as within species comparisons of the amino acid mole percentages of the silks collected in November.

### 3.3. Solid-State NMR (ssNMR) Spectroscopy

We used ^13^C cross-polarization magic-angle spin (CPMAS) solid-state NMR analyses to examine and compare the protein secondary structures of 3 of the *L. hasselti*, *A. keyserlingi* and *N. plumipes* dragline silks collected in May to ascertain whether the amino acid compositions predictably represented protein secondary structures. The silks were not enriched with isotopes since metabolic processing of ^13^C or other isotopes may confound the spectra attained using ^13^C CPMAS ssNMR.

We used 5–10 mg of silk from each spider collected. We performed a pre-assessment using a different set of spiders and found 5–10 mg of unlabeled silk to be adequate to attain reliable NMR spectra. The silk samples were packed in 2.5 mm zirconia MAS rotors with vespel caps. We were careful not to expose the silks to the atmosphere by immediately packing the silks into airtight containers post extraction, and minimizing the exposure to air when packing the sample holders.

The ssNMR spectra were acquired using a Bruker Avance III NMR spectrometer (Bruker Pty Ltd., Melbourne, Australia), with a 16.4 Tesla superconducting magnet operating at 175 MHz and 700 MHz frequencies for the detection of ^13^C and ^1^H isotopes respectively. The 2.5 mm NMR rotor was spun at 30 kHz MAS in a 2.5 mm triple resonance probehead. The ^13^C CPMAS ssNMR spectra were acquired with ^1^H to ^13^C cross-polarization with a 2 ms contact time, SPINAL-64 decoupling for ^1^H decoupling at a field strength of 100 kHz, and a recycle delay of 4 s. Then, 16k–64k transients were co-added to reduce the signal-to-noise ratio. The C=O peak for pure glycine was set to 176 ppm as an external reference for the ^13^C chemical shifts [21]. The width of the alanine C_α_ signal (49.6 ppm) provided insights for determining the overall crystallinity of the ordered domains. Under the acquisition conditions assigned (i.e., 30 kHz MAS), suppression of the ^1^H–^13^C dipolar coupling ensured that only signals from rigid domains could have been detected using our technique.

### 3.4. Tensile Testing

Tensile testing was done on a single thread of dragline silk from each of the 15 spiders collected in May to ascertain whether the amino acid compositions or secondary structures explained the mechanical properties of each of the three species’ silks. To do this, we connected a revolving headframe to the mechanical spool. We attached a 240 mm long × 40 mm wide cardboard (for native silk testing) or plastic (for supercontracted silk testing) strip, which had six 30 mm × 30 mm square holes punched at 10 mm intervals to the headframe. Double sided sticky tape was stuck onto the cardboard at the border of the holes. A single thread was pulled from the spinnerets of an anaesthetized spider and stuck to one of the pieces of sticky tape. The headframe was rotated once ensuring the silk traversed all of the holes and adhered to the tape. The strip was then removed from the headframe and a drop of water based glue applied to the position where the silk was fastened to the cardboard/plastic. Another frame of equal size with identically positioned holes punched into it was placed on top. The two strips were squeezed together with forceps ensuring that they stuck together. We then cut the strip in the regions between the holes perpendicular to the silk thread, leaving six 30 mm × 30 mm frames holding a single thread of silk. The above procedure was repeated for each individual from each of the three species (we accordingly collected 30 frames per species: 6 frames × 5 individuals).

We taped one randomly selected frame of silk collected from each spider frame to a microscope slide and examined and photographed it under 1000× magnification using a polarized light microscope (CKX41, Olympus, Tokyo, Japan) connected to a SPOT Idea 5 Mp digital camera (Spot Imaging Solutions, Sterling Heights, MI, USA). The images were digitized using the program Spot Basic 4.7 (Spot Imaging Solutions, Sterling Heights, MI, USA) and the width of each thread determined as a mean of 12 measurements using the program Image J (NIH, Bethesda, MD, USA). These measurements enabled us to calculate the cross-sectional area of each individual thread used in the ensuing tensile tests.

We performed the following tensile tests under controlled temperature (~25 °C) and humidity (~50% R.H.) in still air within 10 days of silk collection. We performed native state tensile tests on 15 frame-mounted silk samples (3 frames each from 5 individuals) per species. To do this, we placed the 30 mm × 30 mm frames containing a single fiber within the grips of an Instron 5543 tensile testing machine (Instron Machines, Melbourne, Australia) with a resolution to approximately 2 μN. We ensured that the grips held the silks firmly at the upper and lower frame edges. The left and right sides of the frames were cut away and the silks stretched at a rate of 0.1 mm·s^−1^ until the fiber ruptured.

Stress (σ) and strain (ε) were calculated using equations [57]:

(1) $$\sigma =\frac{F}{A}$$

(2) $$\varepsilon =\log_{e}\frac{L}{L_{0}}$$

where F is the force applied to the specimen measured using the program Bluehill 3.0 (Instron Machines, Melbourne, Australia); A is the cross-sectional area of the thread calculated from the thread diameter assuming a constant thread volume; L is the instantaneous length of the fiber at a given extension value measured using Bluehill 3.0; and L_0_ is the original gauge length of the fiber.

Stress versus strain curves were determined for each silk tested by a standard trapezoidal method from which we calculated the following mechanical properties for each specimen: (1) ultimate strength, the stress at rupture; (2) extensibility, the strain at rupture; (3) toughness, the Area under the stress strain curve; and (4) Young’s modulus (stiffness), the slope of the stress–strain curve during its initial elastic phase.

We performed supercontracted tensile tests on 8–10 frame-mounted silk samples (two samples each from four to five individuals) per species to calculate the parameters wet tension and percentage shrink. We submersed the fibers within a perspex water bath while the samples were held within the grips of the tensile testing machine without tension applied. We ascertained how much stress was generated by the restrained silks, then the fibers were relaxed while wet and the percentage shrink calculated as the proportional difference between the pre-shrunk (l_0_) and post-shrunk (l_1_) fiber lengths [29,51]. Since immersion in water is expected to interrupt protein alignment in the amorphous region [11,54], these parameters can be presumed to indicate approximately the amount of amorphous chain alignment in the native silks [51]. We then dried the fibers in air at maximum relaxation, upon which they were subjected to the same tensile testing procedures described for native silks.

We compared the ultimate strength, extensibility, toughness and stiffness in the native and supercontracted states, and the wet tension and percentage shrink in the supercontracted state, between species using Multivariate analysis of variance MANOVA. All statistical analyses were performed after checking the variances for heterogeneity using Levene’s tests.

## 4. Conclusions

We determined herein dragline silk spidroin expression and structural (i.e., β-sheet and _3,10_helix alanine conformations and crystallinity) variations between and within three spider species using HPLC and ssNMR respectively. Our HPLC analysis of amino acid composition and ssNMR analysis of protein secondary structures found conflicting results for *A. keyserlingi* dragline silks collected in May. We tentatively considered this as evidence that silk spinning processes decouple protein secondary structures from spidroin expression. It is nevertheless possible that spidroins other than MaSp1 and MaSp2 also appeared in the dragline silks.

The relatively high proline compositions in *A. keyserlingi* silks from spiders collected in November indicated that it was likely that MaSp2 was predominantly expressed, while the compositions of their silks from those collected in May indicated that it was likely that MaSp1 was predominantly expressed. In May, glycine and alanine within *A. keyserlingi* dragline silk were distributed more toward _3,10_helices/random coil than β-sheets, which contrasted with the conformations within the MaSp1 predominant dragline silks of *N. plumipes* and *L. hasselti*. In the latter species’ silks, glycine and alanine were distributed more toward β-sheets. The greater proportion of β-sheet formations within *N. plumipes* silk is explainable by their high glycine and alanine compositions, with the majority of the alanine forming (A)_n_ sequences and most of the glycine forming (GA)_n_ sequences that conformed into β-sheets.

Our tensile tests and HPLC analyses suggested that spidroin expression had little influence on silk mechanical properties. ssNMR, on the other hand, showed that *L. hasselti* and *N. plumipes* dragline silks had greater alanine β-sheet conformations and crystallinity than did that of *A. keyserlingi*. The greater helical conformations in *A. keyserlingi* dragline silks explained its extensibility. Our finding that alanine compositions did not differ between species while β-sheet conformations differed significantly suggested that “on-the-fly” repositioning of alanine during spinning may be a mechanism for inducing protein secondary structural variations, but this needs verification.

We showed here that while amino acid compositions varied over time in different spiders, decoupling of protein secondary structures from spidroin expression could ensure that functionally effective dragline silks are consistently produced. Our findings improve our understanding of the biological processes that induce dragline silk to vary in chemical, structural and mechanical properties across environments. Such understanding is important if spider dragline silk is to be successfully synthesized commercially.

## Figures and Tables

**Table 1:** Comparisons of: (**a**) The glutamine, serine, glycine, alanine and proline compositions (means with standard errors in parentheses) of *L. hasselti*, *A. keyserlingi* and *N. plumpes* dragline silks from spiders collected in May; (**b**) The glutamine, serine, glycine, alanine and proline compositions (means with standard errors in parentheses) of *L. hasselti* and *A. keyserlingi* dragline silks from spiders collected in November; (**c**) Compositions from dragline silk genetic sequences for *Latrodectus hesperus* (from reference [47]) and *Argiope bruennichi* [48]; * Indicates a statistically significant difference in composition was found between for silks collected in November compared to silks collected in May.

	Amino Acids (Percentage Composition)				
	Glutamine	Serine	Glycine	Alanine	Proline
(**a**) May					
*Argiope keyserlingi*	7.90 (0.88)	7.02 (0.72)	35.39 (1.52)	26.46 (1.87)	4.17 (0.84)
*Nephila plumipes*	6.39 (0.92)	4.77 (0.33)	41.09 (0.45)	30.23 (0.56)	2.71 (0.42)
*Latrodectus hasselti*	9.61 (0.20)	6.78 (0.62)	37.56 (1.67)	26.0 (1.92)	3.13 (0.65)
(**b**) November					
*Argiope keyserlingi*	8.27 (0.84)	7.34 (2.29)	34.61 (6.77)	19.34 (6.76) *	12.53 (1.67) *
*Latrodectus hasselti*	8.99 (0.38)	7.47 (0.46)	32.28 (2.75)	29.55 (2.92)	2.79 (0.65)
(**c**) Sequenced compositions					
*Latrodectus hesperus* MaSp1	6.9	–	33.5	31.1	0.4
*Latrodectus hesperus* MaSp2	11.3	–	42.3	32.7	8.6
*Argiope bruennichi* MaSp1	4.38	5.67	45.05	30.61	0
*Argiope bruennichi* MaSp2	14.50	3.04	38.47	22.51	12.48

## Supplementary Materials

Supplementary materials can be found at www.mdpi.com/1422-0067/17/8/1294/s1.
