# Supplementary material for: Evidence of Decoupling Protein Structure from Spidroin Expression in Spider Dragline Silks
Source: Int J Mol Sci. 2016 Aug 9;17(8):1294. doi: 10.3390/ijms17081294 (PMC5000691; doi:10.3390/ijms17081294)
Supplement: Supplementary file 1 [file ijms-17-01294-s001.pdf]

Supplementary Materials: Evidence of Decoupling Protein Structure from Spidroin Expression in Spider Dragline Silks

Sean J. Blamires, Michael M. Kasumovic, I-Min Tso, Penny J. Martens, James M. Hook and Aditya Rawal

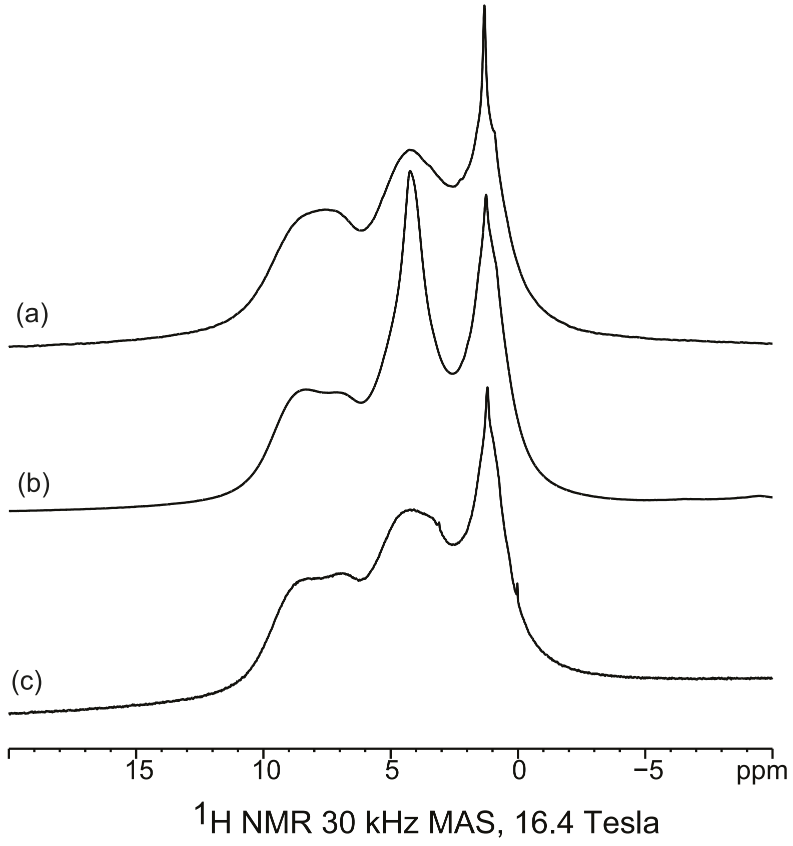

Figure S1. ^{1}H NMR of silk from individual (a) *Argiope keyserlingi*, (b) *Nephila plumipes* and (c) *Latrodectus hasselti*. The spectral peaks at 5 ppm represent the water peak. The peaks are broad and low thus showing that little water had penetrated the structures.

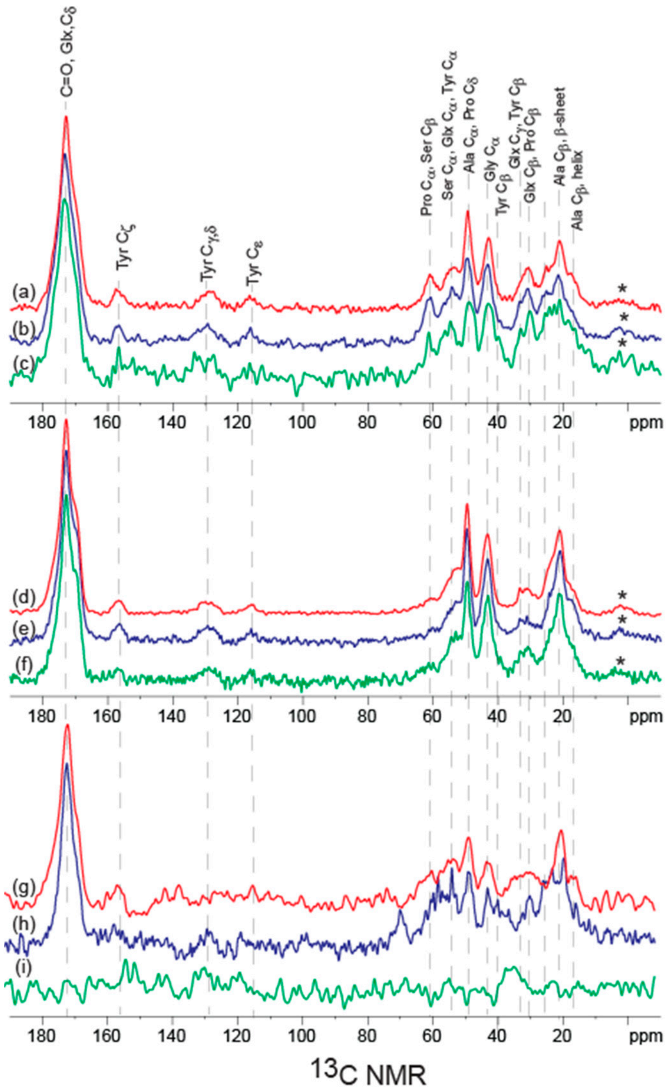

Figure S2. 176 MHz ^{13}C CPMAS ssNMR spectra at 30 kHz for silks from individual spiders of (a–c) *Argiopie keyserlingi*, (d–f) *Nephila plumipes* and (g–i) *Latrodectus hasselti*.

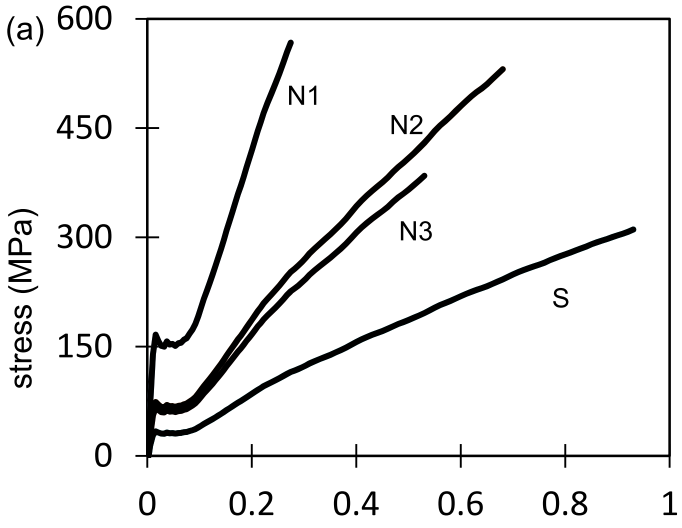

Figure S3. *Cont.*

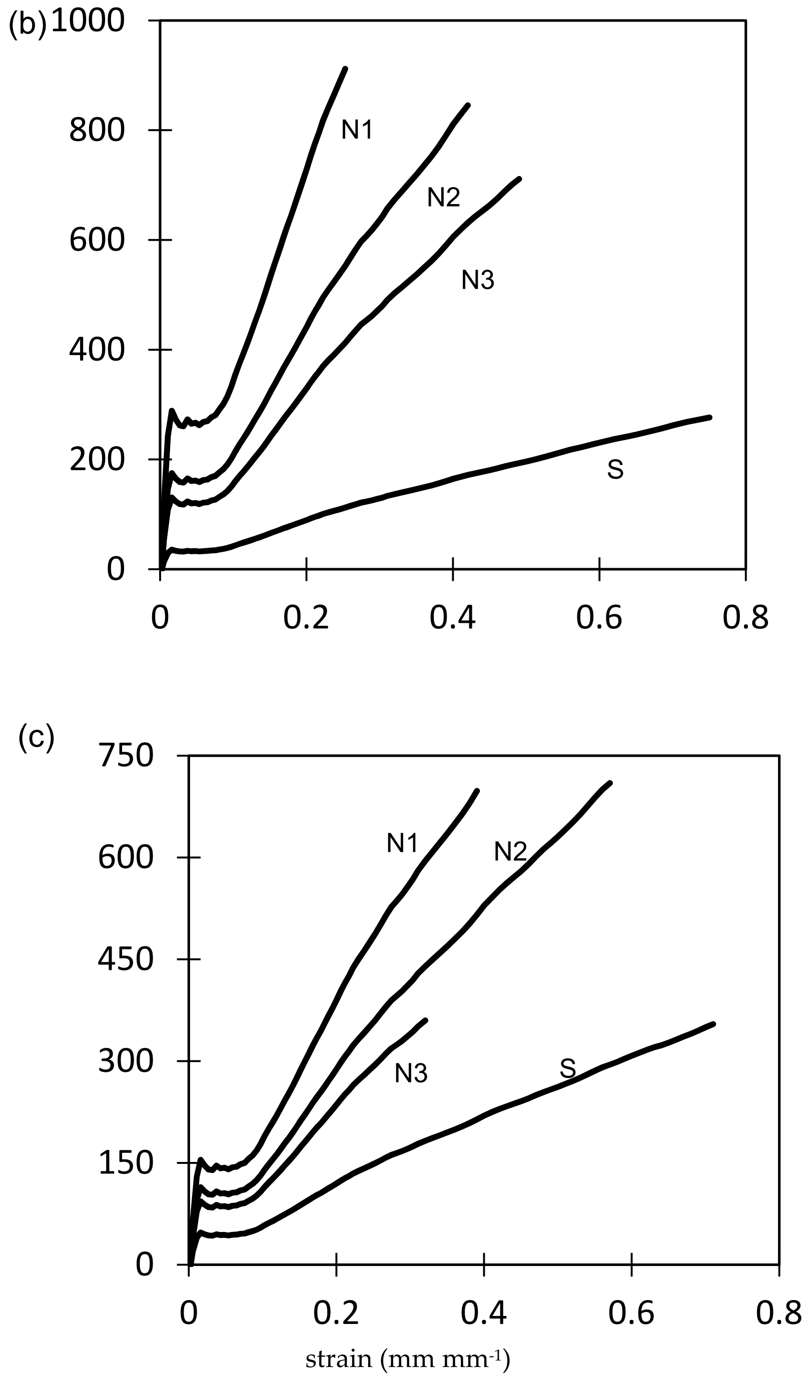

Figure S3. Representative stress-strain curves of (**a**) *A. keyserlingi*, (**b**) *N. plumipes* and (**c**) *L. hasselti* native state dragline silk from three individuals (labelled 'N1', 'N2' and 'N3' respectively) and supercontracted silk from one individual (labelled 'S'). The selected show typical ranges of values. All silks tested were from spiders were collected in May.
